# Better Adjuvants for Better Vaccines: Progress in Adjuvant Delivery Systems, Modifications, and Adjuvant–Antigen Codelivery

**DOI:** 10.3390/vaccines8010128

**Published:** 2020-03-13

**Authors:** Zhi-Biao Wang, Jing Xu

**Affiliations:** National Vaccine and Serum Institute, Beijing Economic-Technological Development Area, Beijing 101111, China; wangzhibiao@live.cn

**Keywords:** vaccine, adjuvant, immune response, immunogenicity

## Abstract

Traditional aluminum adjuvants can trigger strong humoral immunity but weak cellular immunity, limiting their application in some vaccines. Currently, various immunomodulators and delivery carriers are used as adjuvants, and the mechanisms of action of some of these adjuvants are clear. However, customizing targets of adjuvant action (cellular or humoral immunity) and action intensity (enhancement or inhibition) according to different antigens selected is time-consuming. Here, we review the adjuvant effects of some delivery systems and immune stimulants. In addition, to improve the safety, effectiveness, and accessibility of adjuvants, new trends in adjuvant development and their modification strategies are discussed.

## 1. Introduction

Vaccines are effective for preventing diseases. Attenuated and inactivated vaccines have strong immunogenicity, simple preparation processes, and low costs. However, in large-scale applications, because of the long virus culture cycle, the low yield of these vaccines often cannot meet the demand, and the safety may be poor. New vaccines with high purity and good safety are gradually replacing some attenuated and inactivated vaccines in clinical practice. However, these new vaccines have weak immunogenicity and do not induce an effective immune response when used alone [1]. Thus, an adjuvant is used to improve the immune response and increase the efficacy of these vaccines. Adjuvants improve immunogenicity by enhancing antigen presentation to antigen-specific immune cells, resulting in long-term protection against pathogens. Aluminum-containing adjuvants (hereafter referred to as aluminum adjuvants) were the first human vaccine adjuvants approved in clinical use. Although these adjuvants induce strong humoral immunity, they are not equally effective for inducing cellular immunity [2], often making them ineffective against intracellular virus infections [3]. Furthermore, occasionally these adjuvants could cause redness and swelling at the administration site. In addition, the aluminum adjuvants cannot cope with expansion of the vaccine application scope (prophylactic vaccine → prophylactic and therapeutic vaccines) and the development of diversified application methods (intramuscular injection → intramuscular injection, oral administration, nasal drops, etc.). Currently, new and better adjuvants are required for the development of effective vaccines.

A recombinant subunit vaccine (Shingrix^®^) developed using the new Adjuvant Systems 01 (AS01) by GlaxoSmithKline (GSK, Brantford, UK) for preventing shingles (herpes zoster) has been shown to be more effective than the live attenuated vaccine (Zostavax^®^, Merck, Kenilworth, NJ, USA) [4,5,6], demonstrating the key role of adjuvants in vaccine development. In recent years, the process for developing adjuvants has included cross-discipline integration. Preclinical research involves pharmacy (designing and characterization of delivery systems), chemistry (structural modifications based on the structure–activity relationship studies of immune agonists), and biology (evaluation of adjuvant effects). In this review, we focus on the properties of adjuvants and trends in adjuvant research such as the development of various delivery systems as adjuvants, discovery of novel adjuvants, structural modification, application of new small-molecule immune stimulants, and attempts of adjuvant–antigen codelivery.

## 2. Development of New Delivery Systems as Adjuvants

Delivery systems are a common type of adjuvants. Some delivery systems can be used to deliver antigens and/or small-molecule adjuvants (SMAs), whereas others, such as few emulsions, only act as adjuvants and activate the immune response. A list of selected novel delivery systems which also act as adjuvants is provided in Table 1.

### 2.1. Aluminum Salts Adsorb Small-Molecule Adjuvants

It is generally thought that aluminum adjuvants as an antigen delivery system can adsorb antigens and help in their slow release at the injection site, resulting in prolonged immune response [7,8]. However, this mechanism of action of aluminum adjuvants is often challenged. For instance, removal of the injection site two hours after antigen administration had no effect on immune response [9], indicating the existence of different mechanisms associated with the action of aluminum adjuvants. Studies have shown that aluminum adjuvants can either stimulate NLRP3 inflammasomes or induce apoptosis, resulting in the release of dangerous signals, which in turn triggers immune response [10,11,12]. In addition, it has been reported that aluminum adjuvants bind to the cell membrane lipids of dendritic cells (DCs) and alter their structure, thereby stimulating DC cells [13].

The conventional aluminum adjuvants can only induce humoral immunity and weakly induce cellular immunity. Aluminum salts can adsorb low amounts of oppositely charged SMAs through electrostatic interactions [14] when used as an adjuvant delivery system for SMAs. This adsorption can reduce the amount of free SMAs, reducing the risk of SMA-induced cytokine storm and thus enhancing protection. Additionally, these salts can complement the inability of aluminum adjuvants by inducing cellular immunity. Currently, as a representative of such adjuvant systems, AS04 (GSK) which consists of aluminum hydroxide and monophosphoryl lipid A (MPL) has been approved for use in human papilloma virus (HPV) and hepatitis B virus (HBV) vaccines [15]. Similarly, the combination of aluminum and CpG [16] has entered clinical trials as a malaria vaccine [17].

### 2.2. Nanonizationof Aluminum Adjuvants

In recent years, a number of studies have shown that nano-aluminum adjuvants can improve the adjuvant activity of aluminum. For example, compared with traditional aluminum adjuvants, nano-aluminum adjuvants can significantly enhance the immune effect of *Bacillus anthracis* protective antigen and can induce a lower proinflammatory response at the injection site [18]. In an activated rabies virus vaccine, nano-aluminum adjuvants showed better immune enhancement effects than traditional aluminum adjuvants and a few new adjuvants (e.g., AS02, AS03, MF59) [19].

Nano-aluminum adjuvants can absorb more antigen and better present them compared to traditional aluminum adjuvants because of their larger specific surface [20]. Because of the large particle size of traditional aluminum adjuvants, antigen-presenting cells (APCs) can be recruited only at the injection site, whereas the number of APCs in peripheral tissues is low, limiting the immune activation effect of aluminum adjuvants [21]. APCs are abundant in the lymph nodes; however, the positive charge and large particle size of traditional aluminum adjuvants prevent their entry into the lymph nodes. Promoting aluminum adjuvant entry into lymph nodes and then enhancing APC activation using these adjuvants remain difficult. Jiang et al. developed a nano-aluminum adjuvant to overcome these issues using PEG-coated nano-aluminum particles by dispersing aluminum adjuvants via stirring, and then adding PEG to stabilize the particles. Nano-aluminum particles with a negative charge can enter and reside in the lymph nodes; using these particles as adjuvant delivery carriers for CpG can achieve better synergistic effects than traditional aluminum salts [22].

### 2.3. Emulsion Adjuvants

The use of an emulsion as a delivery system has been of specific interest in adjuvant research for a long time. Some emulsion adjuvants available in the market and undergoing clinical trials include oil-in-water (O/W) emulsions MF59 [23] (Novartis, Basel, Switzerland), AS02 and AS03 [24] (GSK), AF03 [25] (Sanofi Pasteur, Lyon, France), SE, MPL-SE, GLA-SE, and SLA-SE (Infectious Disease Research Institute, Seattle, WA, USA) [26,27,28,29], and water-in-oil (W/O) emulsions Montanide ISA720 and Montanide ISA51 [30,31,32] (Seppic’s Montanide).

W/O emulsions usually have a sustained-release effect [33], whereas this is not true for O/W emulsions. For example, when MF59 is used as an adjuvant, the antigen and adjuvant are quickly eliminated from the injection site. Further, the binding of MF59 to antigens has no effect on immune response [34]. However, MF59 can recruit immune cells and promote antigen presentation [35,36]. It has been shown that MF59 can activate MyD88 gene and induce muscle cells at the injection site to release dangerous signals (e.g., ATP), which can activate the downstream immune responses. In addition, apoptosis-associated speck-like protein containing a caspase recruitment domain (CARD) (ASC) is shown to be associated with the MF59 adjuvant effect [37]. Further, emulsions containing squalene, an intermediate product of the cholesterol metabolism in humans, can rapidly reduce the expression of genes related to lipid metabolism in vivo, which results in morphological changes in endoplasmic reticulum (ER) and activation of endoplasmic reticulum stress sensor IRE1α [38].

The emulsion adjuvants can rapidly induce strong humoral immunity, showing stronger effects than aluminum adjuvants in children and elderly individuals (usually with low immune function). Traditional emulsions use surfactants to reduce surface tension, whereas Pickering emulsions are prepared using microparticles. Because Pickering emulsions mimic the fluidity and viscoelasticity of pathogens, the contact area between Pickering emulsions and immune cells is increased, and hence their immune-enhancing effect is better than that of traditional emulsions [39,40]. To develop emulsion adjuvants, Xia et al. mixed all-*trans* retinoic acid with squalene as a flexible core and wrapped it with poly (lactic-co-glycolic acid) to form a rigid outer shell. This unique delivery system enhanced the expression of the DC surface receptor CCR9 when administered by intramuscular injection. In turn, this resulted in antigen uptake and homing of DCs to the mucosal lymph nodes, successfully inducing mucosal immunity [41].

### 2.4. Liposome Adjuvants

Liposomes are another common type of delivery system. The adjuvant system AS01 (GSK) is based on the preparation of common liposomes from dioleoylphosphatidylcholine and cholesterol, and two immunostimulants: the detoxified product MPL, derived from the lipopolysaccharide (LPS) extract of *Salmonella minnesota* strain R595 [42] and QS21, a purified saponin isolated from *Quillajasaponaria Molina* bark [43].

MPL and QS21 in AS01 have a synergistic effect, while cholesterol in liposomes can reduce the hemolytic toxicity of QS21, thus enhancing the safety of adjuvants. QS21 gets accumulated in the lymph after administration, stimulates caspase-1, causing the release of high-mobility group protein B1 (HMGB1), and then activates the TLR4-MyD88 pathway [44]. In addition, QS21 is endocytosed in a cholesterol-dependent manner and gets accumulated in lysosomes. This results in destruction of the lysosomes and releasing of lysosomal enzymes, which in turn activates the downstream immune pathways [45].

As an adjuvant, AS01 can induce strong humoral and cellular immunity. Vaccines containing this adjuvant include Shingrix^®^(GSK, Brantford, UK) for herpes zoster and Mosquirix^®^(GSK, Brantford, UK) for malaria, which were launched in 2017 and 2019, respectively. Another liposome adjuvant, AS015 (AS01 combined with TLR9 activator CpG oligodeoxynucleotide 7909) was used to treat non-small-cell lung cancer and melanoma [46,47].

### 2.5. Virus-Like Particles (VLPs) as Adjuvant Delivery Vectors

Viruses enter human cells in an active or passive manner by interacting with receptor ligands and release their genetic material inside of cells. This process by which viruses function as a delivery vector involves highly efficient targeting. In recent years, the technology used to construct VLPs without viral nucleic acid through genetic engineering approaches has advanced and has been used to develop VLPs as drug delivery vehicles. For instance, hepatitis B core-VLPs were developed and used to load the chemotherapy drug doxorubicin to enhance the anticancer effects and reduce adverse effects [48]. VLPs can be used to package some small molecular adjuvants in the assembly process to achieve adjuvant delivery. For example, packaging of CpG in VLP particles can improve the stability and adjuvant effect of CpG [49]. In addition, VLPs can be used as vaccine adjuvants. For example, papaya mosaic virus coat protein (PapMV CP) expressed by *Escherichia coli* assembles to form VLPs, which can be used as an epitope display system, whereas antigen-fused C-terminal of PapMV CP can trigger a strong immune response [50,51,52].

### 2.6. Microbe-Based Lipid Membrane Delivery Systems

A virosome, another viral-derived delivery vector, can also be used as an adjuvant. The virosome is prepared in vitro using the purified influenza virus envelope protein and lipid. During in vitro assembly, immunostimulants can be rationally designed. Because of its natural outer membrane structure, the virosome has a natural affinity for various immune cells. Vaccines in which virosomes are used as adjuvant include Crucell’s hepatitis A vaccine Epaxal®, Epaxal junior^®^ [53], and seasonal influenza vaccine Nasalflu^®^ [54] (Janssen Vaccines, Berna, Switzerland). Among these, Nasalflu^®^ can cause facial paralysis because of the presence of *E.coli* enterotoxin as a mucosal adjuvant, and hence, this vaccine has been withdrawn from the market [55].

Archaebacteria are a group of microorganisms present in extreme environments, such as under high temperature, high salt, and hypoxia conditions. Their strong survival ability is related to their unique membrane lipid structure, which can be used to prepare archaeosomes. As an adjuvant, archaeosomes have the following advantages over ordinary liposomes: acid resistance (can be used as an oral vaccine adjuvant), high temperature resistance (cold chain transport is not required, and high temperature and pressure can be used for sterilization), and no requirement for cholesterol to stabilize the membrane structure. In addition, archaeosomes are easily taken up by DCs [56]. Furthermore, a few archaeosomes have certain immunostimulatory effects, showing better application prospects than ordinary liposomes [57].

### 2.7. Polymeric Particle Adjuvants

Natural (e.g., chitosan) or synthetic (e.g., poly (lactic-co-glycolic acid)) degradable polymer materials as adjuvant or antigen delivery carriers show good biocompatibility and safety. As an adjuvant, these delivery carriers can granulate free antigens, protect antigens, and enhance antigen uptake by APCs. They can be used to prepare adjuvants with different particle sizes, surface charges, and shapes. Different particles under different pH conditions may have different forms. These properties can be used to load different antigens and induce broad immune cell types. For example, acid-soluble chitosan, after being taken up by DCs, dissolves in the strong lysosomal acid environment, resulting in lysosome rupture via the proton effect, followed by antigen escape and cross-presentation, leading to strong cellular immunity [58].

## 3. Discovery and Structural Modification of Adjuvants

### 3.1. Discovery of Adjuvants Based on Their Targets

In addition to the slow release of antigens and prolonged antigen-stimulating time, the mechanism of action of aluminum adjuvants involves necrosis of cells at the injection site, which results in the release of at least two types of danger signals. One is the release of uric acid, which activates NLRP3 inflammasomes to recruit lymphocytes [11,12] and enhances antigen presentation. The other is DNA release by necrotic cells, which can be recognized by pattern recognition receptors and stimulate the immune response [59].

The trigger of danger signals is an important mechanism of action for some adjuvants [60,61,62]. Among the pattern recognition receptors recognizing danger signals, toll-like receptor (TLR) has been the most commonly studied adjuvant target. Eleven subtypes of TLR have been found in humans, distributed in different parts of the cell and with different natural ligands. The TLRs located on the cell membrane mainly recognize exogenous ligands derived from pathogenic microorganisms, for example, TLR2 combined with TLR6 or TLR1 recognizes diacyl lipopeptides or triacyl lipopeptides; TLR4 recognizes bacterial lipopolysaccharide [63], and the natural ligand for TLR5 is bacterial flagellin [64]. Similarly, meningococcal capsular polysaccharide (CPS) promotes DC maturation [65] by binding to TLR2 and TLR4 [66]. TLRs located on the endosomal membrane mainly recognize nucleic acids. For example, the ligand of TLR3 is double-stranded RNA, such as Poly I: C [67,68], and that of TLR7/8 is single-stranded RNA or oligonucleotide analogues (e.g., resiquimod (R848), imidazoquinolines, and imiquimod), whereas TLR9 recognizes DNA analogs (e.g., CpG) [69]. TLR10 is the only inhibitory TLR, competitive ligands (triacyl lipopeptides) binding with TLR2 and with the effect of specific induction of the anti-inflammatory cytokine IL-1Ra [70].

In addition to TLRs, other intracellular nucleic acid receptors, such as retinoic acid–inducible gene (RIG)-1–like receptors (RLR), nucleotide-binding and oligomerization domain (NOD)-like receptors (NLRs), and stimulator of interferon genes (STING), can recognize some danger signals; STING recognizes cyclic dinucleotide (CDN) analogs (e.g., 2′3′-cGAMP,3′3′-cGAMP,cGMP,cAMP) [71,72] and NLRs recognize muramyl dipeptide (MDP) [73], ATP [74], and uric acid [75]. Some ligands of these receptors have been studied as vaccine adjuvants. The in vitro screening evaluation models of these targets can be used as a powerful tool for adjuvant discovery and evaluation [76]. Table 2 lists selected adjuvant targets, their cellular distribution, and agonists.

Most of these targets are located in the interior of the cells, and it is difficult to reach the target of action with external immunostimulants. In addition to using an adjuvant delivery system, it is necessary to modify the structure of some immunostimulants.

### 3.2. Modification of Adjuvants

Some adjuvants are not suitable for direct use in human vaccines because of their high toxicity (e.g., natural lipopolysaccharides), strong hydrophilic nature (e.g., R848, which cannot easily cross the cell membrane and bind to intracellular receptors), or high degradability (e.g., CDN, which is easily degraded by intracellular phosphodiesterase). In addition to the adjuvant delivery systems, structural modifications of these adjuvants are often required to overcome these limitations. The structures of selected SMAs and their structural modifications are provided in Table 3.

#### 3.2.1. Reducing Adjuvant Toxicity by Chemical Modifications

As a natural ligand of TLR4, bacterial LPS has good adjuvant effect but is highly toxic to humans. Studies of the structure–activity relationship study of LPS showed that its toxicity is mainly caused by three groups on the lipid A molecule: glucosamine disaccharide, two phosphonate groups, and a linear fatty acid [77]. Removing one phosphate group from *Salmonella* R595 lipid A by hydrolysis reduces its toxicity by 100–1000-fold without affecting adjuvant efficacy. Furthermore, removing the 3-*O*-linked acyl group on the disaccharide structure gives 3-*O*-deacyl-4-monophosphoryl lipid A (MPL) [78], a highly effective and proven low-toxic immunoadjuvant [79], which has been used in different vaccines. However, MPL used in these vaccines is the extracted mixture [80], which is not conducive to maintaining product quality. To obtain a single component of MPL, a chemical synthesis method is used to obtain GLA, an MPL analog that retains the hexa-acyl structure and has better adjuvant effects [81]. Based on molecular simulations and docking of GLA and TLR4/MD2 structures, it was predicted that the truncated acyl side chain could enhance the affinity of these compounds to TLR4, which was later confirmed by the synthesis of side-chain-truncated compound SLA [82]. SLA induces less inflammatory cytokines than GLA and may have better safety.

#### 3.2.2. Reducing Adjuvant Toxicity through Synthetic Biology

In addition to chemical synthesis or modification, synthetic biological methods [83,84] can be used to reduce adjuvant toxicity. Methods for introducing or knocking out genes related to lipid A modification in bacteria and reconstructing the biosynthetic pathway of lipid A have been studied. After extraction and separation, MPL analogs with strong adjuvant effects and low toxicity were obtained by in vitro screening. These MPL analogs can be directly used as adjuvants, thus avoiding the influence of subsequent chemical modifications on quality.

#### 3.2.3. Simplifying the Adjuvant Structure and Improving Adjuvant Effect through Structure–Activity Relationship Studies

To minimize the problems associated with chemical synthesis, the structures have been further simplified by synthesizing sugar-free or monosaccharide derivatives of MPL. Through analyses of structure–activity relationships and high-throughput in vitro screening, the MPL sugar-free derivative E6020 was obtained, which showed better immunostimulatory activity and lower toxicity than MPL in various in vitro and in vivo evaluation models [85]. Aminoalkyl glucosyl phosphates are another class of MPL analogs, in which the disaccharide backbone is replaced with a monosaccharide backbone. Some of these compounds showed better activity and safety than MPL [86], includingRC529, which is used as an adjuvant in the hepatitis B vaccine, Supervax^®^(Dynavax, CA, USA).

Resiquimod (R848) is an activator of the TLR7/8 receptor. As an adjuvant, it can promote DC maturation [87] and enhance cellular and humoral immunity [88,89]. Similar to other small-molecule agonists of TLR7/8, R848 is highly water-soluble, which leads to its rapid dispersion after being injected into the body, weakening its effect on promoting the maturation of antigen-presenting cells at the administration site, which is not ideal for its role as an adjuvant. Furthermore, because of the fast dispersion rate, it increases the risk of adverse effects. In addition, the water solubility nature of R848 is not conducive for its entry into the cell and for activating the intracellular receptor, TLR7/8. To enhance lipid solubility and reduce the dispersion rate of these small molecules, fatty acid chains are typically added through chemical modifications, which enhances their membrane permeability [90]. For example, 3M-052 is synthesized by adding C18 long-chain fatty acid through chemical modification. It shows enhanced adjuvant effects compared to R848 [91]. Imiquimod is an analog of R848. The compound SM-360320 was synthesized by simplifying and replacing groups of imiquimod. This compound exhibits better oral bioavailability and can be used as an adjuvant for oral vaccines [92,93].

#### 3.2.4. Improving Bioavailability through Modification

STING is a recently discovered intracellular adaptor protein. The natural ligand of STING is CDN. Binding of CDN to STING stimulates the downstream NF-κB pathway, which promotes type 1 interferon secretion. Thus, CDN can be used as a vaccine adjuvant [94,95,96,97,98]. Among the natural CDNs, 2′3′-cGAMP has the strongest activity; however, it gets easily degraded by intracellular enzymes and has low stability. One O of the phosphate group in 2′3′-cGAMP was replaced with S to obtain thiol 2′3′-cGsAsMP, which showed increased affinity towards STING. Furthermore, because of its resistance to enzymatic hydrolysis, the half-life of thiol 2′3′-cGsAsMP was increased by approximately 20-fold and the stimulatory activity by 10-fold [99]. Similarly, thiol modifications of c-di-GMP also showed an increased half-life and stimulatory activity [100].

## 4. Adjuvant–Antigen Codelivery

**Figure 1:**
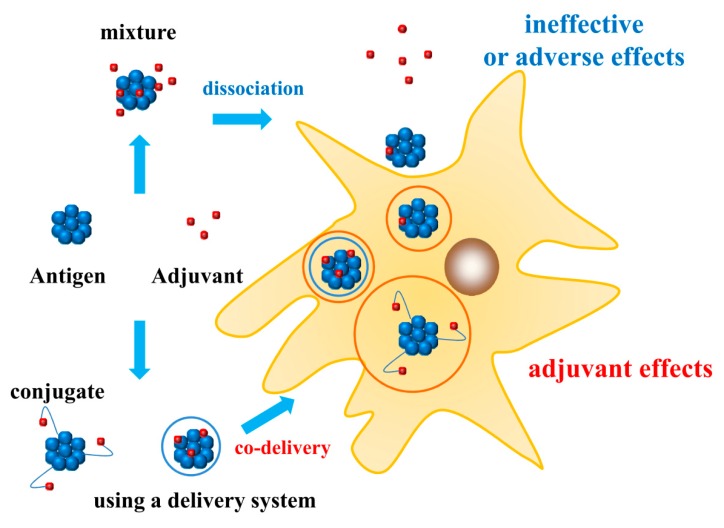
Different interactions between antigens and adjuvants may induce different effects.

Simple mixing of adjuvants with antigens results in their dissociation after entering the body. Not only are free adjuvants rapidly degraded [101], but also the amount of adjuvants entering the cells is reduced, resulting in weak immune stimulation. Furthermore, the free adjuvants may induce autoimmunity, provoking an immune response against the host proteins [102]. Hence, codelivery of adjuvants with antigens using an appropriate system not only makes the stimulation effect of the adjuvant more precise and powerful, but also reduces their off-target effects, making the vaccine safer. Two common approaches can be used for codelivery of adjuvants and antigens: using a delivery system to package the antigen and adjuvant and covalently coupling the antigen with the adjuvant. The different effects may be induced by different interactions between antigens and adjuvants, as shown in Figure 1.

### 4.1. Adjuvant–Antigen Codelivery Using a Delivery System

In addition to the delivery of a single adjuvant, delivery systems can be used for codelivery of adjuvants and antigens. For example, in the immunization of mice with liposome-encapsulated CpG and antigen OVA, the codelivery system effectively increased the secretion of OVA-specific IgG2a and IFN-γ compared to that by administration of antigen alone or a simple mix of CpG and OVA [103]. Liposomal-encapsulated CpG and antigen HER-2/neu increased antigen-specific IFN-γ secretion by 100-fold compared with administration of antigen alone. Furthermore, the immune-enhancement effect of only adjuvant or a simple mix of adjuvant and antigen was shown to be insignificant [104].

Using meningococcal CPS nanoparticles as antigen, in vitro experiments were performed to evaluate the effect of antigen–adjuvant coincubation on DC cell maturation. The maturation of DC cells was better when coincubated with antigen and adjuvant than that for incubation with nanoparticles or adjuvant alone, although the stimulation effect of different adjuvants varied [105]. However, these results need to be further verified using in vivo experiments.

Further, another kind of antigen–adjuvant codelivery is realized by self-adjuvanted action of antigens. Because some antigens themselves have good adjuvant effect, they can achieve considerable immune response without the need of additional immunostimulatory adjuvants. For example, some whole-cell inactivated vaccines have strong adjuvant effects due to their intact pathogen structure; however, their safety is questionable. Thus, using an antigen delivery system ensures slow release of antigens and helps in achieving good effectiveness and safety by prolonged stimulation of immune response from small antigen doses [106].

### 4.2. Covalent Coupling of Protein Adjuvants to Antigens by Gene Fusion

Protein adjuvants, such as cytokines, bacterial flagellin, and heat shock protein (HSP), are mostly codelivered with antigens by gene fusion. For example, the cytokine IL2 or GM-CSF is fused with pneumococcal surface protein A. Compared to pneumococcal surface protein A alone, the antigen–adjuvant conjugate significantly improves the immune response [107]. The fused protein between *Helicobacter pylori* urease B and IL2, expressed in the lactobacillus system, has been shown to effectively increase the level of anti-*H. pylori* urease B antibody [108]. Compared to hyaluronic acid (HA) alone, the fusion protein between IL2 and HA can be better taken up by APCs, resulting in a higher T-cell response and significantly enhanced immune response [109].

HSPs are generally produced under stress. These proteins have certain immunomodulatory effects and can be used as vaccine adjuvants. An adjuvant codelivery system produced by the fusion of HSP-70 and HIV-1 p24 proteins stimulates stronger cellular immunity than a simple antigen–adjuvant mix [110]. Using the gene fusion method, mouse Hsp70 has been fused with tumor antigen MAGE-A1 to obtain an adjuvant–antigen codelivery vaccine. Compared with those without adjuvant or simple adjuvant–antigen mix, the codelivery vaccine can significantly delay tumor growth and improve the survival time of tumor-bearing mice [111]. The fusion protein between human papilloma virus 16 (HPV16) mE6/mE7 and TBHSP-70 proteins has been studied as a tumor therapeutic vaccine and showed good antitumor effects [112].

As an adjuvant, TLR5 ligand flagellin can also achieve codelivery with antigen by gene fusion. The C-terminus of flagellin was fused with the HA1 fragment of A/Solomon Islands/3/2006 H1N1 to obtain the adjuvant–antigen codelivery vaccine VAX125. A Phase-I clinical trial showed that small doses (1μg) of the adjuvant presented good safety and efficacy, whereas high doses (3–8 μg) caused flu-like symptoms associated with C-reactive protein [113]. By changing the coupling site between flagellin and HA, the fusion protein vaccine VAX128B (flagellin N-terminal fused with HA1) and VAX128C (flagellin N-terminus and C-terminus fused with HA1) were obtained, which showed the same immunogenicity but greater safety [114].

### 4.3. Enzyme-Catalyzed Covalent Coupling of Adjuvants and Antigens

The gene fusion method may affect the integrity and immunogenicity of antigens. With the development of protein modification technologies, non-gene fusion methods have been used for coupling of protein adjuvants and antigens. Sortase A is a class of enzyme found in gram-positive bacteria and is associated with protein modification through recognition of specific protein sequences (LPXTG motifs); thus, it can be used for site-directed modification of proteins [115,116,117,118]. Influenza M2e peptide was conjugated with PapMV coat-protein (CP) by Sortase A to obtain an antigen–adjuvant complex with good immune response [119]. In another study, the PapMV CP and full-length influenza nucleoprotein were covalently coupled with Sortase A, and the resulting complex induced stronger cellular and humoral immunity [120]. The TLR2 agonist FSL-1 was coupled to the surface of group A streptococcal recombinant protein using Sortase A. The antigen-specific IgG induced by the codelivery system was 1000-fold higher than that of the simple mixture [121].

### 4.4. Chemical Coupling of Small Molecular Adjuvants and Antigens

Unlike protein-based adjuvants, SMAs are often coupled to antigens by chemical coupling. The Ag85B protein of *Mycobacterium tuberculosis* fused with HspX antigen (AH) as an antigen, TLR receptor agonist poly (I:C) modified by arabinogalactan as an adjuvant (AG-P), and antigen–adjuvant codelivery vaccine AH-AG-P were obtained by chemical coupling. The codelivery system showed a better immune response than the simple mix of adjuvant–antigen [122]. Cross presentation of the OVA antigen was significantly enhanced by chemical coupling of a CpG molecule [123]; however, this cross-presentation did not increase with the number of coupled CpG molecules. For HA antigen displayed on the surface of ferritin and then coupled with TLR9 agonist CpG or TLR7/8 agonist 3M012 [124], the dosage of chemically coupled adjuvants was reduced by 5000-fold while achieving the same immune enhancement effect compared to noncoupled adjuvants [125].

## 5. Conclusions

Vaccines play an important role in preventing infectious diseases; however, no effective vaccines against AIDS, tuberculosis, or many other diseases have been developed. The successful development of vaccines relies on a more comprehensive and detailed understanding of their pathogenic mechanisms as well as on the development of vaccine technology [126]. Adjuvants are important parts of vaccines. However, a limited number of adjuvants and the limited understanding of their mechanism of action prevent the rational design of vaccines. Appropriate use of adjuvants can modulate the body’s response to antigens, such as speeding up antibody production, prolonging protection time, and reducing adjuvant-induced adverse effects. Herein, we introduced various means aimed at making the adjuvant effect more precise, safer, and more effective. The scope of application of adjuvants will gradually expand in the future, where the target of vaccine adjuvants will be that of antiviral and antitumor drugs. Multiple studies have reported that compounds with adjuvant effects often have antiviral or antitumor pharmacological activities [127,128,129]. Adjuvants should be developed not only for vaccines, but also for evaluating their potential antiviral or antitumor pharmacological activities. A platform for in vitro screening of adjuvants and evaluation of their targets would facilitate these studies.

Some natural products such as QS21 have favorable adjuvant effects. However, because of its complex structure, QS21 cannot be fully synthesized using chemical synthesis methods. It can only be extracted from plants at present, greatly limiting the production capacity of vaccines using this adjuvant. On one hand, the structure can be simplified using the structure–activity relationship to reduce the difficulty of synthesis. On the other hand, the emergence of new technologies such as synthetic biology may provide another source of complex adjuvants. For example, reconstructing biosynthetic pathway of LPS analogs in bacteria through gene knockout and insertion has been successfully used to prepare MPL [130].

The focus has shifted from traditional preventive vaccines to therapeutic vaccines, among which cancer therapeutic vaccines have garnered considerable attention. However, this may make the adjuvant system more complicated. As most tumor-specific antigens are the only aberrantly expressed autoantigens, the immunogenicity is weak and it is difficult for the antigens alone to produce a therapeutic effect because of the immunosuppressive microenvironment of tumor cells [131]. Using such vaccines, it is often difficult to achieve the desired results with existing single adjuvants, and a combination of multiple adjuvants is considered an appropriate solution. However, because of differences in the adjuvant sites of combined adjuvants, a more complex adjuvant delivery system is required to effectively activate their respective targets. Furthermore, the combination of multiple adjuvants may increase safety risks. Further studies are needed to investigate the desired effects and avoid the adverse effects of combined adjuvants.

Adjuvants and antigens have never been isolated, and a uniform design may be useful for these applications. Using bionic theory for the integrated design of adjuvants and vaccines [132,133] and developing combinations of adjuvant and antigen more similar to “exogenous microbes” will improve the activation of the body’s immune response. However, this bionic design will make the vaccine components appear complex and difficult to control. Another trend is to simplify the vaccines; for example, the antigen itself can be used as a delivery carrier, with the adjuvant inserted on the surface of the antigen by coupling to form an extremely simplified antigen–adjuvant complex [134,135,136]. However, this method may result in missing or blocking of some epitopes during the modification process. No adjuvant or delivery system is a panacea. Rational design of adjuvants requires integration of multiple disciplines and long-term collaborations between researchers from different fields.

## Figures and Tables

**Table 1:** Selected novel delivery systems that act as adjuvants.

Adjuvants	Classifications	Components	Mechanisms or Receptors
AS04	Aluminum salt-based combined adjuvant	MPL + Alum	TLR4
Alum + CpG	Aluminum salt-based combined adjuvant		TLR9
MF59	O/W emulsion	Tween 80, span85, squalene	MyD88, ASC
AS02	O/W emulsion	MPL, QS21, AS03	TLR4
AS03	O/W emulsion	Tween 80, α-tocopherol, squalene	IRE1α
AF03	O/W emulsion	Span80, polyoxyethylene cetyl-stearylether, mannitol, squalene	Immune cell recruitment
SE	O/W emulsion	Glycerol, phosphatidylcholine, squalene	Immune cell recruitment
MPL-SE	O/W emulsion	MPL, SE	TLR4
GLA-SE	O/W emulsion	GLA, SE	TLR4
SLA-SE	O/W emulsion	SLA, SE	TLR4
Montanide ISA-720	W/O emulsion	Mannide monooleate, squalene	Depot effect, immune cell recruitment
Montanide ISA-51	W/O emulsion	Mannide monooleate, mineral oil	Depot effect, immune cell recruitment
AS01	liposome	MPL, QS21, DOPC, cholesterol	TLR4, immune cell recruitment
AS015	liposome	CpG, AS01	TLR4, TLR9, immune cell recruitment
Virosome	Microbe-based lipid membrane delivery systems		Promote antigen presentation
Archaeosomes	Microbe-based lipid membrane delivery systems		Promote antigen presentation

**Table 2:** Adjuvant targets, their cellular distribution, and agonists.

Adjuvant Targets	Cellular Distribution	Agonists
TLR1	Cell membrane	Triacyl lipopeptides
TLR2	Cell membrane	Triacyl lipopeptides, diacyl lipopeptides, CPS
TLR3	Endosomal membrane	Double-stranded RNA analogs (e g., Poly I: C)
TLR4	Cell membrane	MPL analogs (e.g., GLA, SLA, RC529, E6020)
TLR5	Cell membrane	Bacterial flagellin
TLR6	Cell membrane	Diacyl lipopeptides
TLR7	Endosomal membrane	Single-stranded RNA analogs (e.g., resiquimod (R848), imidazoquinolines, imiquimod, and 3M-052)
TLR8	Endosomal membrane	Single-stranded RNA analogs (e.g., resiquimod (R848), imidazoquinolines, imiquimod, and 3M-052)
TLR9	Endosomal membrane	DNA analogs (e.g., CpG)
STING	Endoplasmic reticulum	Cyclic dinucleotide analogs (e.g., 2′3′-cGAMP, 3′3′-cGAMP, cGMP, cAMP),
NLR (e.g., NLRP3, NOD1, NOD2)	Cytoplasm	Muramyl dipeptide (MDP), ATP, uric acid

**Table 3:** Structures and structural modifications of selected small-molecule adjuvants.

Structure	Molecule	Characteristic
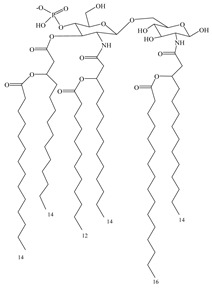	MPL	Extracted mixture, difficult to guarantee consistency
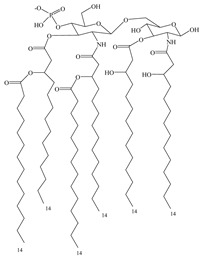	GLA	Chemically synthesized MPL analog that retains the hexa-acyl structure and has better adjuvant effects
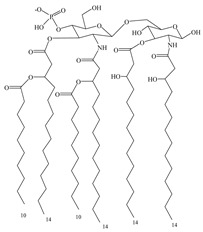	SLA	Side-chain-truncated compound of GLA, which showed higher affinity for TLR4
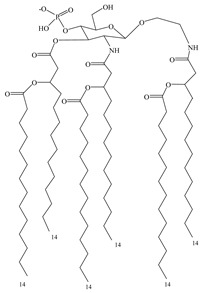	RC529	MPL derivative in which the disaccharide backbone is replaced with a monosaccharide backbone
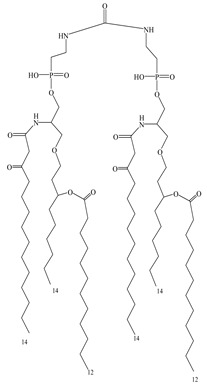	E6020	MPL sugar-free derivative
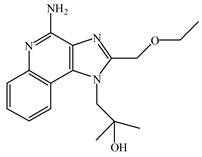	R848	TLR7/8 agonist
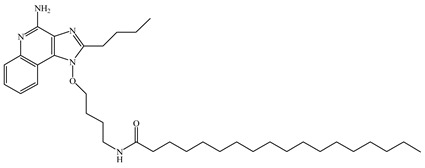	3M-052	With the addition of a long fat chain, the permeability of membrane was enhanced and the effect was better than that of R848
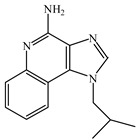	Imiquimod	TLR7/8 agonist
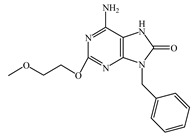	SM-360320	Synthesized by simplifying and replacing groups of imiquimod, which presented better oral bioavailability
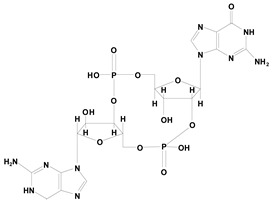	2′3′-cGAMP	STING agonist
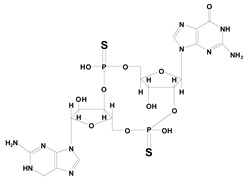	2′3′-cGsAsMP	Resistant to enzymatic hydrolysis
